# Relationship between body mass index‐to‐thigh circumference ratio and incident hypertension among community‐dwelling persons

**DOI:** 10.1002/jgf2.70048

**Published:** 2025-07-21

**Authors:** Ryuichi Kawamoto, Kikuchi Asuka, Daisuke Ninomiya, Masanori Abe, Teru Kumagi

**Affiliations:** ^1^ Department of Community Medicine Ehime University Graduate School of Medicine Toon Ehime Japan

**Keywords:** body mass index, hypertension, thigh circumference

## Abstract

**Background:**

The incidence of hypertension is increasing worldwide. Recent studies have suggested that smaller thighs are a disadvantage for health and survival, but the association of body mass index (BMI) adjusted for thigh circumference (BMI‐to‐thigh ratio) and hypertension remains uncertain.

**Methods:**

This study included 788 men (mean age 69 ± 10 years) and 975 women (mean age 69 ± 10 years) from a rural village. The relationship between BMI‐to‐thigh ratio and hypertension was examined using both cross‐sectional (baseline, *N* = 1763) and cohort data (follow‐up, *N* = 433). Logistic regression models were used to evaluate the BMI‐to‐thigh ratio as a significant predictor of hypertension.

**Results:**

Both the cross‐sectional and cohort data demonstrated that higher BMI‐to‐thigh ratios were associated with an increased prevalence and incidence of hypertension. In the cohort data, participants in the fourth quartile (odds ratios [OR]: 2.58, 95% confidence interval [CI]: 1.15–5.80) and third quartile (OR: 2.54, 95% CI: 1.24–5.20) of the BMI‐to‐thigh ratio had significantly higher ORs compared with the first quartile. In the cross‐sectional data, the optimal BMI‐to‐thigh ratios cutoff values for predicting hypertension were 51 (sensitivity: 71.1%; specificity: 54.3%) for men and 51 (sensitivity: 65.0%; specificity: 70.6%) for women. In the cohort data, the optimal BMI‐to‐thigh ratios were 50 (sensitivity: 73.2%; specificity: 52.3%) for men and 47 (sensitivity: 70.9%; specificity: 51.2%) for women.

**Conclusions:**

The findings suggest that the BMI‐to‐thigh ratio is a useful screening tool for incident hypertension.

## Graphical abstract

Receiver‐operating characteristics curve analysis to identify optimal BMI‐to‐thigh ratio to distinguish subjects with hypertension in the cross‐sectional and cohort data.

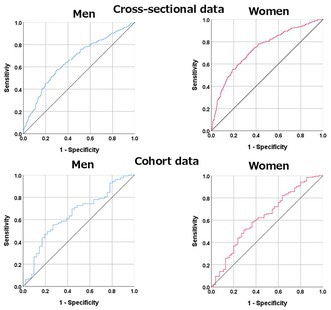

## 1 INTRODUCTION

Hypertension is a major global contributor towards disability and mortality and is recognized as a critical preclinical condition that significantly increases the risk of cardiovascular diseases (CVD), including those affecting the heart, brain, and kidneys, as well as overall mortality.^1^, ^2^ Its onset is closely linked to several modifiable lifestyle factors, such as high salt intake, diets rich in saturated and trans fats, insufficient consumption of fruits and vegetables, physical inactivity, tobacco use, excessive alcohol intake, and obesity.^1^

In adults, thigh circumference—which is strongly associated with physical function (e.g., gait speed)^3^—is often used as a proxy for muscle mass and lower body adiposity. Thigh circumference, which reflects quadriceps muscle mass and peripheral subcutaneous fat,^4^ has been shown to have protective associations with long‐term blood pressure (BP) regulation, arterial stiffness, and the risk of developing hypertension.^5^ Additionally, a smaller thigh circumference has been linked to diabetes, with a stronger association observed among individuals with a body mass index (BMI) of less than 25 kg/m^2^.^6^ However, thigh circumference is significantly influenced by body size, such as BMI. The correlation coefficients between thigh circumference and BMI were reported to be 0.70 for men and 0.69 for women.^6^ Notably, BMI is also recognized as a marker for hypertension.^7^, ^8^ Consequently, the association between larger BMI and decreased risk of hypertension^5^ observed before adjusting for thigh circumference may reflect shared associations with obesity‐related indicators.^9^ Thus, BMI adjusted for thigh circumference, referred to as the BMI‐to‐thigh ratio, has emerged as a novel biomarker and is considered an excellent predictor of incident hypertension. Despite extensive research on these topics, few studies have explored the relationship between BMI‐to‐thigh ratio and hypertension in community‐dwelling individuals.

The aim of this study is to investigate the association between BMI‐to‐thigh ratios and hypertension in rural, community‐dwelling Japanese individuals.

## 2 METHODS

### 2.1 Participants

The present study utilized data from the Nomura study conducted over a three‐year period from April 1, 2014 to December 31, 2017. The participants were community‐dwelling individuals recruited through an annual municipal health screening provided for residents aged 20 years and older at the Nomura Health and Welfare Center in Nomura‐cho, Seiyo‐city, located in the Ehime prefecture, Japan.^10^ The research complied with ethical norms and followed the guidelines outlined in the Declaration of Helsinki, with each participant providing written consent after being fully informed. Approval for this study was granted by the ethics committee at Ehime University School of Medicine (Institutional Review Board Approval Number: 1402009).

In 2014, 1832 community‐dwelling participants (818 men and 1014 women) aged 22–95 were initially registered. Participants with missing data, particularly on BP and thigh circumference (*N* = 69), were excluded from the study. The final dataset comprised 1763 participants (788 men and 975 women). In this longitudinal data, the development of hypertension was investigated over a three‐year period in 643 individuals after excluding 512 men and 608 women with preexisting hypertension (defined as clinical diagnosis of hypertension diagnosis or current use of antihypertensive medication as described in the following section). After excluding cases with missing follow‐up data, a final analysis was conducted on 433 participants.

### 2.2 Evaluation of risk factors

Trained staff conducted standardized face‐to‐face interviews using a questionnaire to collect information on participants' current health status, including medical history and medication use. The BMI (kg/m^2^) was calculated by dividing the body weight (BW) in kilograms by the square of the body height (BH) in meters. Thigh circumferences (m) were measured with an anthropometric tape (25‐204; Clover, Osaka, Japan). Thigh circumference was measured just below the gluteal fold on both legs, and the mean of the two was used in the analysis. The BMI‐to‐thigh circumference ratio (BMI‐to‐thigh ratio) was computed by dividing the BMI by thigh circumference. Smoking status was defined by multiplying the number of cigarette packs smoked per day by the number of years of smoking (pack‐years). Participants were categorized into four groups: never smoked, former smokers, light smokers (less than 30 pack‐years), and heavy smokers (30 pack‐years or more). Drinking habits were evaluated using the Japanese unit, equivalent to 22.9 g of ethanol, with participants classified as never drinkers, occasional drinkers (less than 1 unit/day), and daily drinkers (moderate: less than 2 units/day; heavy: 2 units/day or more). Systolic blood pressure (SBP) and diastolic blood pressure (DBP) were measured twice on the right upper arm of participants in a seated position after a minimum rest period of 5 min, using an appropriately sized cuff and an automatic oscillometric BP recorder (BP‐103i; Colin, Aichi, Japan), and the average of both measurements was calculated. Blood samples were obtained in the morning following an overnight fast of at least 11 h. These samples were used to evaluate several parameters, such as triglycerides (TG), high‐density lipoprotein cholesterol (HDL‐C), low‐density lipoprotein cholesterol (LDL‐C), hemoglobin A1c (HbA1c), serum creatinine (Cr), and serum uric acid (SUA). The estimated glomerular filtration rate (eGFR) was assessed utilizing the Chronic Kidney Disease–Epidemiology Collaboration (CKD–EPI) equations, which were adjusted with a Japanese coefficient, denoted as eGFR_CKD–EPI_: male, Cr ≤0.9 mg/dL: 141 × (Cr/0.9)^−0.411^ × 0.993^age^ × 0.813; Cr >0.9 mg/dL: 141 × (Cr/0.9)^−1.209^ × 0.993^age^ × 0.813; and female, Cr ≤0.7 mg/dL: 144 × (Cr/0.7)^−0.329^ × 0.993^age^ × 0.813; Cr >0.7 mg/dL: 144 × (Cr/0.7)^−1.209^ × 0.993^age^ × 0.813.^11^

### 2.3 Criteria for clinical diagnosis of hypertension

Hypertension was classified based on the use of antihypertensive medication and/or an SBP of 140 mmHg or higher and/or a DBP of 90 mmHg or higher, as defined by the 2020 International Society of Hypertension Global Hypertension Practice Guidelines.^12^

### 2.4 Statistical analysis

Statistical analyses were performed using IBM SPSS Statistics version 26 (SPSS Japan, Tokyo, Japan). Unless otherwise specified, mean values are presented alongside their standard deviations. Due to nonnormal data distributions, TG and HbA1c are reported as medians with interquartile ranges, and log‐transformation was applied prior to analysis. Participants were stratified into two groups based on hypertensive status, and differences were evaluated using the Student's *t*‐test for continuous variables and the chi‐square test for categorical variables. To identify predictors of hypertension, the area under the receiver‐operating characteristics (ROC) curve was calculated for each variable. The ROC curve depicts sensitivity (true positive rate) against 1 − specificity (false positive rate) for the respective markers. The reference ROC curve represents the line connecting the points (0,0) to (0,1) and (0,1) to (1,1). Generally, an ROC curve falls between these boundaries, with the area under the ROC curve (AUC) serving as a summary statistic reflecting the overall diagnostic accuracy across varying test thresholds. Predictive values were determined using the formulas: positive predictive value = sensitivity/(sensitivity + (1 − specificity)) and negative predictive value = specificity/((1 − sensitivity) + specificity). To identify the optimal cutoff points for hypertension, the Youden index was computed as sensitivity + specificity − 1, with the value yielding the maximum Youden index taken as the optimal cutoff. To assess the influence of baseline BMI‐to‐thigh ratio and various confounding factors (including gender, age, smoking status, drinking status, presence of CVD, TG, HDL‐C, LDL‐C, use of antidyslipidemic medication, HbA1c, use of antidiabetic medication, eGFR, and SUA) on hypertension prevalence in a cross‐sectional data and hypertension incidence in a cohort data, multiple logistic regression analysis was performed. In cases where independent variables showed a high correlation (*r* ≥ 0.6), indicating multicollinearity, the correlated variable (e.g., BMI) was excluded from the multivariate analysis. Statistical significance was defined as *p* < 0.05.

## 3 RESULTS

### 3.1 Baseline characteristics of study subjects categorized by presence of hypertension in the cross‐sectional data

This cross‐sectional data included 1763 participants at baseline. The study population consisted of 788 men (mean age 69 ± 11 years) and 975 women (mean age 69 ± 10 years). Table 1 presents the baseline characteristics of the participants, categorized by hypertension status. Men accounted for 42.9% of the normotension group and 45.7% of the hypertension group. Compared with the nonhypertension group, the hypertension group exhibited significantly higher values for age, BMI, BMI‐to‐thigh ratio, drinking status, prevalence of CVD, SBP, DBP, use of antihypertensive medication, TG levels, use of antidyslipidemic medication, HbA1c, use of antidiabetic medication, and SUA. Conversely, smoking status, HDL‐C levels, and eGFR were significantly lower in the hypertension group.

**TABLE 1:** Baseline characteristics of subjects according to hypertension status in the cross‐sectional data.

Baseline characteristics (*N* = 1763)	Nonhypertension (*N* = 643)	Hypertension (*N* = 1120)	*p* *
Male gender, *n* (%)	276 (42.9)	512 (45.7)	0.274
Age (years)	65 ± 12	71 ± 8	**<0.001**
Body mass index (kg/m^2^)	21.8 ± 2.9	23.4 ± 3.2	**<0.001**
Thigh circumference (m)	0.439 ± 0.040	0.440 ± 0.039	0.657
Body mass if‐thigh circumference ratio	50 ± 5.0	53 ± 5.2	**<0.001**
Smoking habit (never/past/light/heavy [%])	70.8/15.6/6.1/7.6	72.3/20.4/1.8/5.4	**<0.001**
Drinking status (never/occasional/light/heavy [%])	49.8/26.3/8.6/15.4	50.2/20.7/10.3/18.8	**0.022**
Cardiovascular disease, *n* (%)	28 (4.4)	89 (7.9)	**0.004**
Systolic blood pressure (mmHg)	121 ± 12	144 ± 15	**<0.001**
Diastolic blood pressure (mmHg)	72 ± 8	81 ± 10	**<0.001**
Antihypertensive medication (%)	0	769 (68.7)	**<0.001**
Triglycerides (mg/dL)	82 (61–112)	91 (69–129)	**<0.001**
HDL cholesterol (mg/dL)	67 ± 18	64 ± 16	**0.001**
LDL cholesterol (mg/dL)	121 ± 29	119 ± 30	0.129
Antidyslipidemic medication (%)	87 (13.5)	296 (26.4)	**<0.001**
Hemoglobin A1c (%)	5.6 (5.4–5.8)	5.7 (5.5–6.0)	**<0.001**
Antidiabetic medication (%)	34 (5.3)	117 (10.4)	**<0.001**
Estimated GFR (mL/min/1.73 m^2^/year)	76.1 ± 11.0	69.7 ± 12.3	**<0.001**
Serum uric acid (mg/dL)	5.1 ± 1.3	5.4 ± 1.4	**<0.001**

*Note*: Data presented are mean ± standard deviation. Data for triglycerides and hemoglobin A1c are skewed and presented as median (interquartile range) values, and they were log‐transformed for analysis. Bold values indicate significance (*p* < 0.05).

Abbreviations: GFR, glomerular filtration ratio; HDL, high‐density lipoprotein; LDL, low‐density lipoprotein.

* *p*‐Value: Student's *t*‐test for the continuous variables or the *χ* ^2^ test for the categorical variables.

### 3.2 Results of the ROC curve analysis to identify optimal obesity indices to discriminate participants with hypertension in the cross‐sectional data

**FIGURE 1:**
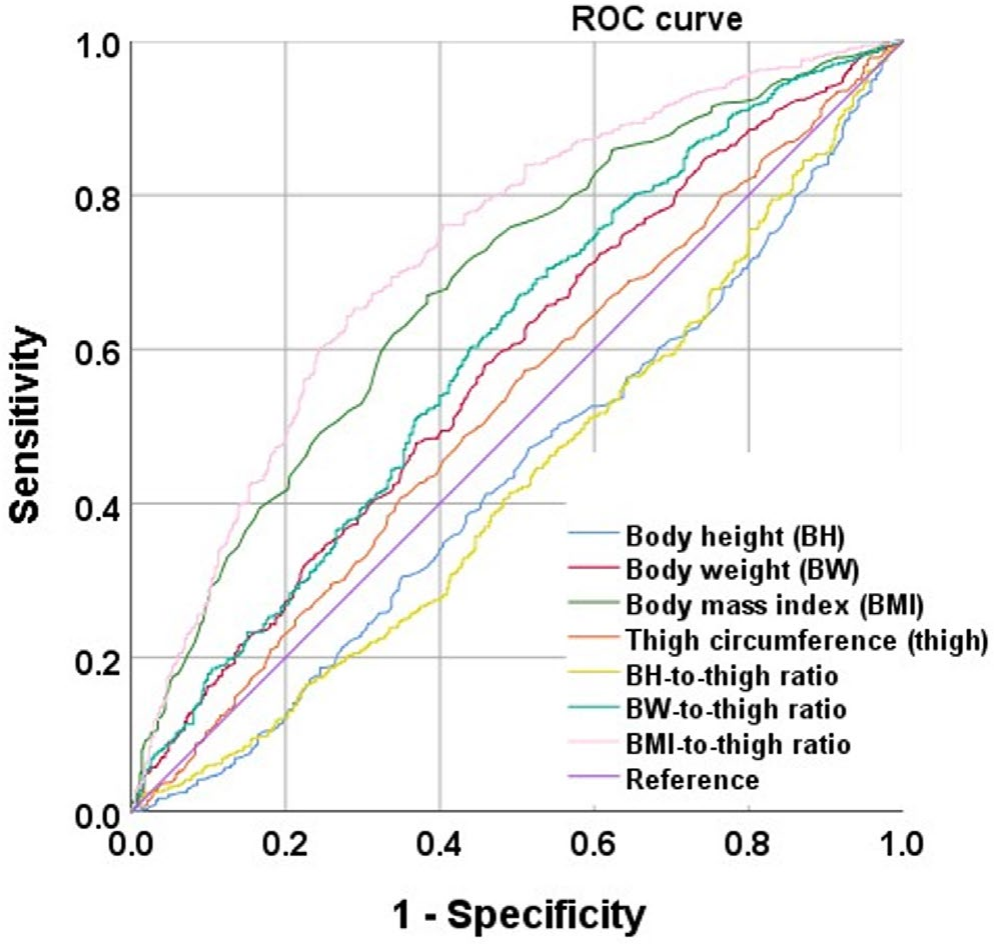
ROC curve analysis to identify optimal obesity indices to discriminate subjects with hypertension in the cross‐sectional data. Body height (BH) (AUC: 0.420, 95% CI: 0.392–0.447), body weight (BW) (AUC: 0.553, 95% CI: 0.525–0.581), body mass index (BMI) (AUC: 0.656, 95% CI: 0.630–0.683), thigh circumference (thigh) (AUC: 0.506, 95% CI: 0.478–0.534), BH‐to‐thigh ratio (0.434, 95% CI: 0.406–0.462), BW‐to‐thigh ratio (AUC: 0.572, 95% CI: 0.543–0.600), and BMI‐to‐thigh ratio (AUC: 0.705, 95% CI: 0.679–0.730).

Figure 1 shows the ROC curve for BH, BW, BMI, thigh circumference, BH‐to‐thigh ratio, BW‐to‐thigh ratio, and BMI‐to‐thigh ratio for each hypertension in participants using ROC analyses. In the cross‐sectional data, BMI‐to‐thigh ratio as well as BMI and BW‐to‐thigh showed significantly predictive ability for hypertension, with BMI‐to‐thigh ratio (AUC: 0.705, 95% confidence interval [CI]: 0.679–0.730) showing the strongest predictive ability.

### 3.3 Results of the ROC curve analysis to identify optimal obesity indices to distinguish subjects with hypertension in the cross‐sectional and cohort data

**FIGURE 2:**
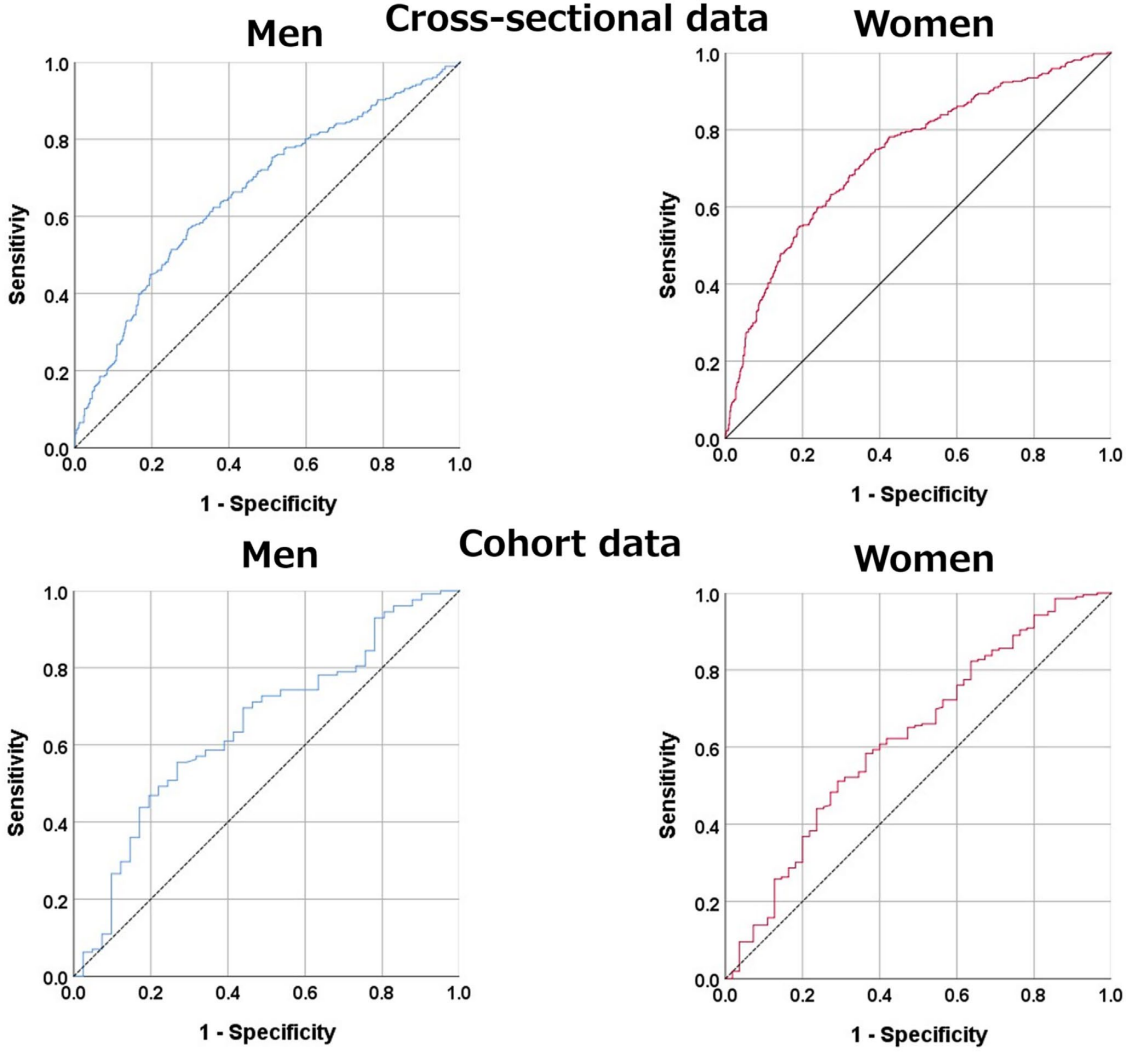
ROC curve analysis to identify optimal BMI‐to‐thigh ratio to distinguish subjects with hypertension in the cross‐sectional and cohort data. Area under the receiver‐operating characteristic curve (AUC) values (95% CI) for BMI‐to‐thigh ratio to discriminate subjects with hypertension in the cross‐sectional and cohort data.

Figure 2 and Table 2 present the AUC for hypertension in both men and women as determined by ROC analyses. In the cross‐sectional data, the BMI‐to‐thigh ratio demonstrated a significant capacity to predict hypertension in both men (AUC: 0.663, 95% CI: 0.623–0.704) and women (AUC: 0.736, 95% CI: 0.703–0.768). Similarly, in the cohort data, the BMI‐to‐thigh ratio significantly predicted the onset of hypertension in both men (AUC: 0.647, 95% CI: 0.551–0.744) and women (AUC: 0.627, 95% CI: 0.541–0.712).

**TABLE 2:** Best cutoff values of BMI‐to‐thigh ratio to predict hypertension in the cross‐sectional and cohort data.

	*N*	AUC (95% CI)	*p*	Cutoff value	Sensitivity	Specificity	PPV	NPV	Accuracy
Cross‐sectional data *N* = 1763									
Men	788	0.663 (0.623–0.704)	**<0.001**	≥51	71.1%	54.3%	74.3%	50.3%	65.2%
Women	975	0.736 (0.703–0.768)	**<0.001**	≥51	65.0%	70.6%	78.5%	54.9%	67.1%
Cohort data *N* = 433									
Men	169	0.647 (0.551–0.744)	**0.005**	≥50	73.2%	52.3%	33.0%	85.9%	57.4%
Women	264	0.627 (0.541–0.712)	**0.004**	≥47	70.9%	51.2%	27.7%	87.0%	55.3%

*Note*: Bold values indicate significance (*p* < 0.05).

Abbreviations: AUC, area under the receiver‐operating curve; NPV, negative predictive value; PPV, positive predictive value.

### 3.4 Best cutoff values of BMI‐to‐thigh ratio to predict hypertension in the cross‐sectional and cohort data

As shown in Table 2, in the cross‐sectional data, the optimal BMI‐to‐thigh ratio thresholds for predicting hypertension were determined to be 51 for men (sensitivity: 71.1%, specificity: 54.3%) and 51 for women (sensitivity: 65.0%, specificity: 70.6%) as shown in Table 3. In the cohort data, the optimal cutoff values were 50 for men (sensitivity: 73.2%, specificity: 52.3%) and 47 for women (sensitivity: 70.9%, specificity: 51.2%).

**TABLE 3:** Odds ratios and 95% CI for hypertension of subjects according to quartiles of BMI‐to‐thigh ratio in the cross‐sectional and cohort data.

Cross‐sectional data *N* = 1763	*N*	Odds ratio (95% CI)			
		BMI‐to‐thigh ratio			
		Quartile 1	Quartile 2	Quartile 3	Quartile 4
Men	788	38–49	49–52	52–55	55–73
Women	975	37–47	47–51	51–55	55–85

	*N* = 441	*N* = 440	*N* = 442	*N* = 440	*p*‐Value
Hypertension prevalence	167 (37.9%)	266 (60.5%)	330 (74.7%)	357 (81.1%)	**<0.001**
Nonadjusted	Reference	1.46 (1.06–2.01)	2.81 (2.07–3.82)	7.06 (5.19–9.59)	**<0.001**
Gender‐ and age‐adjusted	Reference	1.44 (1.04–1.99)	2.32 (1.69–3.19)	4.91 (3.56–6.77)	**<0.001**
Multivariable‐adjusted^a^	Reference	1.38 (0.99–1.93)	2.09 (1.51–2.91)	4.23 (2.98–6.01)	**<0.001**

Cohort data *N* = 433	*N*	Quartile 1	Quartile 2	Quartile 3	Quartile 4
Men	169	38–48	48–50	50–53	53–66
Women	264	38–45	45–48	48–51	51–68

	*N* = 108	*N* = 113	*N* = 104	*N* = 108	*p*‐Value
Hypertension incidence	14 (13.0%)	18 (15.9%)	30 (28.8%)	34 (31.5%)	**0.001**
Nonadjusted	Reference	1.13 (0.63–2.04)	2.43 (1.27–4.63)	3.09 (1.54–6.17)	**0.008**
Gender‐ and age‐adjusted	Reference	1.01 (0.55–1.84)	2.18 (1.13–4.21)	2.47 (1.18–5.14)	**0.009**
Multivariable‐adjusted ^a^	Reference	0.99 (0.53–1.86)	2.54 (1.24–5.20)	2.58 (1.15–5.80)	**0.008**

*Note*: Bold values indicate significance (*p* < 0.05).

Abbreviation: CI, confidence interval.

^a^ Multivariable‐adjusted for gender, age, smoking status, drinking status, presence of cardiovascular disease, triglycerides, HDL cholesterol, LDL cholesterol, use of antidyslipidemic medication, HbA1c, use of antidiabetic medication, eGFR, and serum uric acid. Data for triglycerides and Hb A1c were log‐transformed for analysis.

### 3.5 Odds ratios and 95% confidence intervals for hypertension by quartile of BMI‐to‐thigh ratio in the cross‐sectional and cohort data

To assess whether the BMI‐to‐thigh ratio is independently associated with hypertension after accounting for potential confounders, a multiple logistic regression analysis was conducted. Hypertension was used as the dependent variable, while various confounding factors were adjusted (Table 3). Both the cross‐sectional and cohort studies revealed that higher BMI‐to‐thigh ratios were associated with a higher prevalence and incidence of hypertension. In the cohort data, the baseline BMI‐to‐thigh ratio was a significant predictor of incident hypertension. In particular, the fourth quartile (odds ratio [OR]: 2.58, 95% CI: 1.15–5.80) and the third quartile (OR: 2.54, 95% CI: 1.24–5.20) of the BMI‐to‐thigh ratio showed higher odds ratios (ORs) than the first quartile (reference group).

## 4 DISCUSSION

In this cross‐sectional and cohort data, we found that a higher BMI‐to‐thigh ratio is significantly associated with higher prevalence and incidence of hypertension, independent of confounding factors. Moreover, the optimal BMI‐to‐thigh ratio thresholds for predicting hypertension were 51 (men and women) in cross‐sectional data, and 50 (men) and 47 (women) in cohort data. These data reinforce the importance of thigh circumference and BMI as a modifiable determinant of cardiometabolic risk in both genders. To the best of our knowledge, no epidemiologic studies have quantified the link between BMI‐to‐thigh ratio and incidence and prevalence of hypertension in community‐dwelling Japanese persons.

Obesity and hypertension are intricately linked, with obesity estimated to be a contributing factor to 65%–78% of primary hypertension cases.^13^ Assessing the distribution of excess adipose tissue using anthropometric tools is a fundamental approach that provides valuable insights for identifying individuals at cardiovascular risk (e.g., BH, BW, BMI, and thigh circumference).^14^ Among these tools, BMI is widely utilized globally due to its simplicity.^8^ Epidemiological studies show that obesity, as defined by BMI, is a well‐established risk factor for cardiometabolic morbidity and mortality.^15^ Weight gain, together with overweight and obesity (defined as a BMI of 25 to <30 kg/m^2^ and ≥30 kg/m^2^, respectively), contributes to an increased risk of hypertension through various pathophysiological mechanisms.^16^, ^17^, ^18^, ^19^ However, BMI has significant limitations as a predictive factor: It does not account for fat distribution and cannot distinguish between fat mass and fat‐free mass.^20^, ^21^ Moreover, BMI thresholds for defining being overweight and obesity may inaccurately assess hypertension risk across different ethnic groups. For example, Asians are predisposed to metabolic disorders, such as diabetes, dyslipidemia, and hypertension, even at BMI values and weight ranges considered normal by Western standards.^22^

A small thigh circumference may also reflect reduced skeletal muscle mass and/or subcutaneous fat in the thighs, both of which have been strongly associated with increased risk of hypertension in individuals aged 40 years and older,^5^ diabetes in participants aged 30–79 years,^6^ as well as CVD^23^ and total mortality^23^, ^24^ in both adult men and women. Shi et al.^5^ showed that compared with the lowest thigh circumference tertile group, the risk of hypertension was significantly lower in the highest tertile group, both in overweight individuals, defined as 24 kg/m^2^ ≤ BMI < 28 kg/m^2^ (OR 0.68; 95% CI: 0.59–0.79) and obese individuals, defined as BMI ≥28 kg/m^2^ (OR 0.51; 95% CI: 0.38–0.70). BMI has been identified as an important modifier in this context, particularly among men with a BMI above 25 kg/m^2^. Obesity exacerbates hypertension risk, while a reduction in thigh skeletal muscle mass further amplifies this risk. This interplay may partially explain our observation of a stronger association between small thigh circumference and increased hypertension risk, particularly in overweight and obese individuals.^5^ Notably, our data found that thigh circumference alone was not associated with hypertension. Instead, BMI adjusted for thigh circumference emerged as an excellent predictor of hypertension development.

The mechanisms by which obesity leads to hypertension are multifaceted, involving overactivation of the sympathetic nervous system,^25^ stimulation of the renin‐angiotensin‐aldosterone system,^26^ dysregulation of adipose‐derived cytokines,^27^ insulin resistance,^28^ and structural as well as functional changes in the kidneys.^29^ In addition, research has shown that low levels of thigh subcutaneous fat are also linked to impaired lipid and glucose metabolism, regardless of high abdominal fat, while higher levels of thigh subcutaneous fat are associated with glucose profiles^30^ and improved lipid.^31^ Thigh subcutaneous fat has been found to have a negative correlation with fasting glucose, postload glucose, and insulin resistance.^32^ Additionally, thigh muscle mass area also has been inversely related to the development of insulin resistance in the future.^33^ Consequently, impaired glucose and lipid metabolism, which contributes to vascular dysfunction, may explain the association between low thigh circumference and an elevated risk of high BP.

The major strength of this data is that the analysis was conducted after adjusting for thigh circumference in both cross‐sectional and cohort studies. Additionally, numerous potential covariates were considered in the analysis to minimize the impact of confounding risk factors. However, several potential limitations should be acknowledged. First, the cross‐sectional and cohort design limits the ability to establish causal relationships between the BMI‐to‐thigh ratio and BP measurements. Second, reliance on a single measurement of SBP and DBP may introduce misclassification bias. Third, the data could not fully account for the presence of transient hypertension or white‐coat hypertension. Fourth, it was not possible to entirely eliminate the influence of underlying diseases on the outcomes. Furthermore, the potential effects of medications for hypertension, dyslipidemia, and diabetes on the findings could not be excluded. Finally, a small sample size might have affected statistical power and the precision of estimated associations. In addition, the study participants were limited to those who attended annual health checkups and therefore, the findings may not be applicable to the general population. As a result, the demographic characteristics and recruitment sources of the participants may impose limitations on the generalizability of the findings.

## 5 CONCLUSIONS

This data demonstrates that the BMI‐to‐thigh ratio is strongly and inversely associated with the incidence of hypertension in the general population. Although the underlying mechanism behind this relationship remains unclear, it appears to be independent of confounding factors. Further analysis of longitudinal data from this data may offer more definitive insights into this association.

## AUTHOR CONTRIBUTIONS

**Ryuichi Kawamoto:** Conceptualization; methodology; data curation; funding acquisition; investigation; formal analysis; writing – review and editing; writing – original draft. **Kikuchi Asuka:** Conceptualization; investigation. **Daisuke Ninomiya:** Conceptualization; investigation. **Masanori Abe:** Conceptualization; methodology. **Teru Kumagi:** Supervision; conceptualization.

## FUNDING INFORMATION

This research received partial support from a Grant‐in‐Aid for Scientific Research from the Foundation for Development of Community (2024). No additional external funding was received. The funders had no involvement in the data design, data collection and analysis, decision to publish, or manuscript preparation.

## CONFLICT OF INTEREST STATEMENT

The authors affirm that the study was carried out without any commercial or financial connections that may be seen as a possible conflict of interest in the cross‐sectional and cohort study. Dr. Kawamoto, Ryuichi is an Editorial Board member of JGFM Journal and a co‐author of this article. To minimize bias, they were excluded from all editorial decision‐making related to the acceptance of this article for publication.

## ETHICS STATEMENT

Ethics approval statement: The research complied with ethical norms and followed the guidelines outlined in the Declaration of Helsinki, with each participant providing written consent after being fully informed. Approval for this study was granted by the ethics committee at Ehime University School of Medicine (Institutional Review Board Approval Number: 1402009).

Patient consent statement: None.

Clinical trial registration: None.
